# Leveraging black soldier fly larvae for sustainable poultry waste management and high-value nanomaterial recovery: a path towards circular economy

**DOI:** 10.3389/fbioe.2026.1707445

**Published:** 2026-08-11

**Authors:** Prasad M. Govindaiah, Diksha P. Gourkhede, Rituparna Banerjee, Naveena B. Maheswarappa, B. H. Niranjan, Judy Lalthanmawii, M. Kiran, S. Wilfred Ruban

**Affiliations:** 1 ICAR- Krishi Vigyan kendra, Indi, University of Agricultural Science, Dharwad, India; 2 Division of Veterinary Public Health, ICAR-Indian Veterinary Research Institute, Bareilly, India; 3 ICAR-National Meat Research Institute, Hyderabad, Telangana, India; 4 Department of Livestock Products Technology, Veterinary College, Bangalore, Karnataka, India; 5 ICAR-Indian Veterinary Research Institute, Bareilly, India

**Keywords:** bioconversion, black soldier fly larvae (BSFL), chitin nanomaterials, circular economy, poultry waste management, sustainable waste valorization

## Abstract

The escalating global demand for poultry products has led to a commensurate increase in poultry waste, posing significant environmental and economic challenges. Conventional waste management strategies, including rendering, composting, landfilling, incineration, and biogas, often suffer from limitations such as high operational costs, greenhouse gas emissions, and incomplete resource recovery, thereby failing to offer truly sustainable solutions. This review critically examines the potential of Black Soldier Fly (*Hermetia illucens*) larvae (BSFL) as an innovative and eco-friendly approach to poultry waste management, aligning with circular economy principles. We delve into the biological characteristics and bioconversion capabilities of BSFL, highlighting their efficiency in transforming diverse poultry wastes, including hatchery by-products, abattoir waste, and on-farm residues, into valuable biomass rich in protein and lipids, and a nutrient-dense frass/powder suitable for use as an organic fertilizer. This manuscript elucidates the mechanisms of BSFL-mediated waste degradation, nutrient upcycling, and pathogen reduction, underscoring significant reductions in waste volume (up to 50%), nitrogen (30%–50%), and phosphorus (61%–70%), alongside minimal greenhouse gas emissions compared to traditional methods. Emerging nanobiotechnological applications further enable the derivation of high-value nanomaterials, such as chitin nanocrystals and nanocomposites, from BSFL by-products for uses in medicine, agriculture, and advanced materials. The use of nanobiotechnology in the BSF technique can replace the conventional method of waste management with the high throughput novel techniques, Furthermore, this review explores optimization of BSFL rearing systems, economic viability, and the multifaceted environmental benefits, such as odor mitigation and enhanced biosecurity. By converting a problematic waste stream into valuable resources, BSFL technology not only addresses critical waste management issues but also contributes to sustainable animal feed production and agricultural practices. This comprehensive analysis underscores the transformative potential of BSFL bioconversion as a cornerstone for sustainable poultry waste valorization, paving the way for a more circular and resilient agri-food system.

## Graphical Abstract

**Figure:**
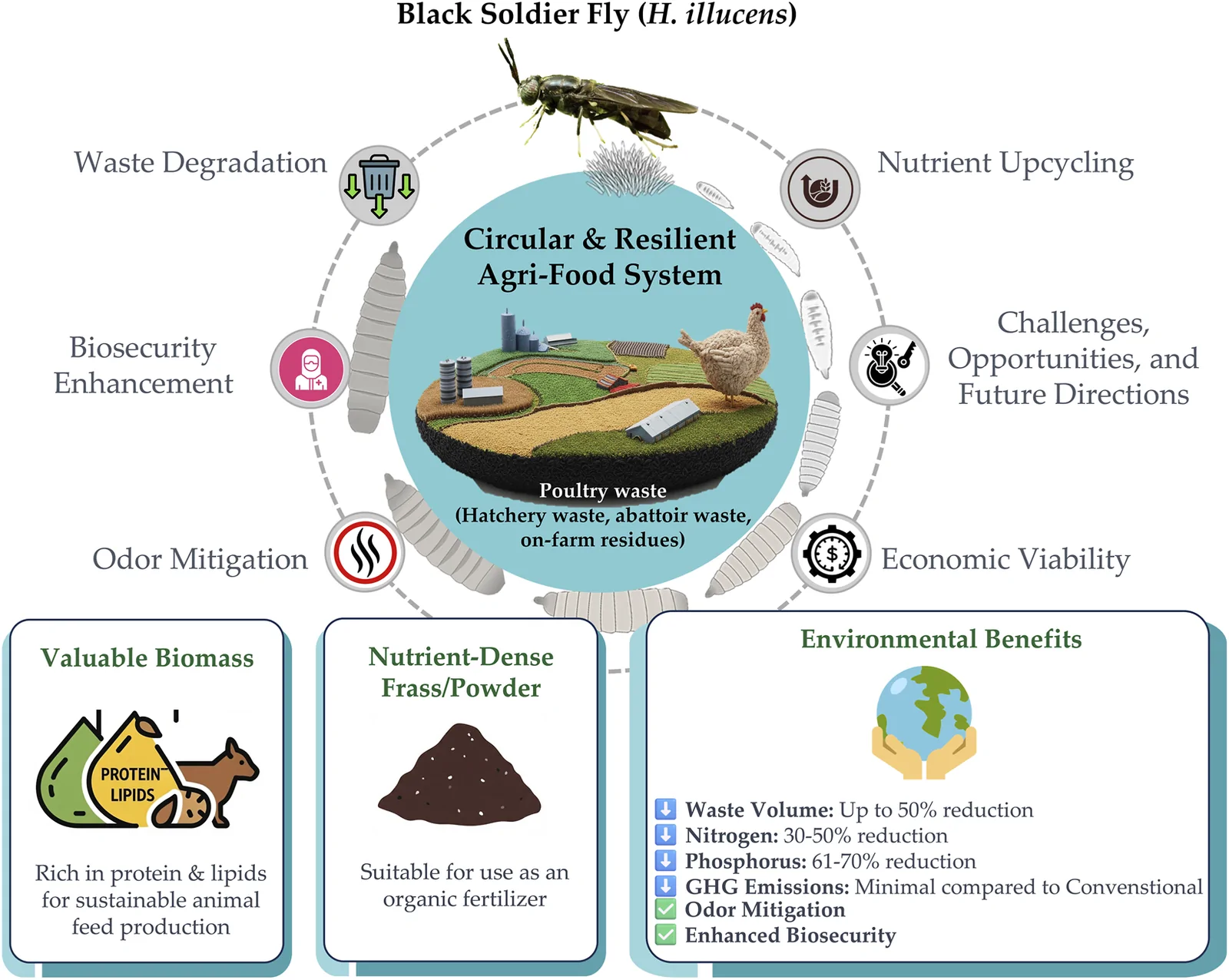

## Highlights

- BSFL convert poultry waste into protein, lipids, and organic fertilizer.
- Reduces volume by 50%, nitrogen by 30%–50%, phosphorus by 61%–70%.
- Minimal greenhouse gas emissions vs. traditional methods.
- Reduces pathogens, enhancing biosecurity.
- BSFL nanobiotech: poultry waste to high-value nanomaterials.

## 1 Introduction

Being one of the most effective animal husbandry practices, poultry farming provides substantial nutritional security to the whole global population. Globally, the poultry sector continues to grow and industrialize with advances in farming, breeding, and processing practices (FAO, 2023). Global poultry meat production significantly increased from 9 to 140 million tonnes between 1961 and 2023, while egg production surged from 15 to 97 million tonnes in the same period. Poultry meat comprised nearly 40% of worldwide meat output. Notably, world egg production has grown by 150% over the past 3 decades. During the last 3 decades, the world’s poultry industry has undergone a significant transformation in its organizational structure and mode of operation, evolving from small backyard farming to a substantial poultry sector with integrated participants (APEDA, 2022; FAO, 2024). The poultry market is experiencing strong growth, projected to increase from $384.95 billion in 2024 to $410.98 billion in 2025, demonstrating a compound annual growth rate (CAGR) of 6.8% (Rybicka et al., 2024). With a steady increase in global demand for poultry, several intensive farming practices are coming into play, which might contribute to significant environmental footprint.

The improper usage of poultry waste, such as hatchery waste, slaughter waste (feathers, intestine, heads, and feet), and poultry farm waste such as litter waste (cage and deep) can cause a several environmental, biological, economical, and industrial issue (Ashayerizadeh et al., 2017) (Table 1). Therefore, management of poultry waste is crucial for safeguarding the environment, public health, and safe poultry production (Rahman et al., 2022). These challenges necessitate orienting the efforts in poultry waste management towards minimizing environmental impact. The lack of regulations regarding waste management in most of the developing countries is causing environmental contamination, surface, and ground water pollution (Ferronato and Torretta, 2019). Poultry waste management majorly comprises techniques such as rendering, composting, landfilling, and incineration (Mozhiarasi and Natarajan, 2025). However, these commercially available techniques have some drawbacks such as, requirement of special equipment, high usage of electricity and emission of gas and odor in rendering, land area and emission of greenhouse gases in landfilling and composting (El Seoud and Mady, 2019; Nordahl et al., 2020). Therefore, more sustainable methods are required to find an economically-viable and environment friendly way for waste management. In this context, insect farming using organic waste is an emerging area to combat the aforementioned issues related to recycling of organic waste.

**TABLE 1:** Comparative analysis of waste (animal origin) management methods: Environmental, economic, and health-related limitations.

Method	Environmental limitations	Economic limitations	Health-related limitations	References
Rendering	Wastewater generation, odor and VOC emissions, energy consumption	High capital and operational costs, efficiency dependent on separation	Risk of disease transmission if rendered products used as animal feed	Leiva et al. (2018), Müller et al. (2023)
Composting	Odor emissions (ammonia, VOCs), land requirements	Extended processing time, variability in nutrient content	Potential for incomplete pathogen inactivation and regrowth	Ayilara et al. (2020)
Landfilling	Leachate contamination of water resources, greenhouse gas emissions (methane)	Land scarcity, potential decrease in property values	Potential for disease transmission through pests and contaminated water	Sampat et al. (2018), Siddiqua et al. (2022)
Incineration	Air pollution (particulate matter, dioxins, heavy metals), hazardous ash disposal	High capital and operational costs, energy consumption for operation and pre-treatment	Potential health risks from air pollutants and ash contamination	Council (2000), Groff et al. (2014)
Combustion	Air pollutant emissions (NOx, SO2, CO, PM), ash management	Need for fuel preparation (drying, pelletizing), variable energy efficiency	Potential health risks from air pollutants and ash	Council (2000)
Biogas Production	Sensitivity to substrate variability, hydrogen sulfide production, digestate management	Technological limitations in digester efficiency, costs of scrubbing and digestate handling	Potential for pathogens in digestate if not properly treated	EESI (2017), Saleh and Hassan (2021), Werkneh (2022)
Biodiesel Production	Energy-intensive lipid extraction, environmental footprint of production process	Low lipid content in waste, feedstock availability and consistency	Indirect health impacts through environmental pollution from production processes	Chauhan et al. (2011), Adewale et al. (2015), Toldrá-Reig et al. (2020)

Utilization of poultry waste for insect farming is a bioremediation method which helps in generating protein-rich feed and organic fertilizer (Surendra et al., 2020). Over the decades, the black soldier fly larvae (BSFL) has been studied as a bioconversion technology for organic waste management (Gold et al., 2020). BSF is a two-pronged biological tool that can salvage the waste on one hand, and resource recycling on the other. Larvae of BSF are commercially produced on agricultural waste streams and convert these into animal body protein and fat (Purkayastha and Sarkar, 2022). Feed resource generated by recycling through the larvae can be comparable to the conventional feed resources that are currently employed in animal and poultry feeding in India. Larval meal might be a feasible alternative protein source for the partial replacement of soya and fish meal. The nutritional profile of the wild BSFL indicates its potential to serve as a cheap and sustainable substitute protein source (Nyakeri et al., 2017). Globally, especially in developing countries, an integrated system of poultry rearing is at sociological and environmental risk, where the integrator models have failed in the proper disposal of waste in cages as well as deep litter at the field level. Along with this, the model suffered a lot from the house fly menace. So, the above lacunae in the poultry sector need to be dealt with new technology like use of BSFL.

Beyond producing protein-rich feed and organic fertilizer, recent advances in nanobiotechnology are unlocking higher-value applications from BSFL-processed poultry waste. These include the derivation of chitin-based nanomaterials for antimicrobial scaffolds, precision nano-fertilizers from frass, and conductive bionanocomposites for biosensing and flexible electronics. Such innovations represent a “nanorefinery” paradigm, further strengthening the circular economy by transforming low-value by-products into functional materials for medicine, agriculture, and advanced technologies.

Recent trends highlight growing interest in using Black Soldier Fly Larvae (BSFL) for organic waste management. Existing reviews demonstrate BSFL’s effectiveness in processing agricultural waste (Beyers et al., 2023), kitchen waste (Mahmood et al., 2021), livestock manure (dairy and poultry) (Rehman et al., 2023), and other organic waste (Mahmood et al., 2021; Amrul et al., 2022). However, these reviews lack detailed insights into poultry waste management, a significant global challenge. This review addresses the global burden of poultry waste, evaluates conventional disposal methods, and highlights their limitations. It also compares the efficacy of BSFL in treating poultry waste against these methods. A key contribution of this review is its focus on advancing the circular economy through BSFL-based poultry waste management, emphasizing benefits such as environmental sustainability, resource recovery, and pathogen reduction. Also, the nanotechnology in the BSF novel method is the new area to work on and future studies can focused on the utilization of waste using the BSF technique by incorporating the nanotechnology. This method will help in replacing the conventional method with the novel high through put techniques.

The current review has included studies published between 2010 and 2024, sourced from academic databases such as PubMed, Web of Science, and Google Scholar. We excluded ResearchGate from the final search to ensure peer-reviewed sources, though it was initially consulted for grey literature. Keywords used included “Black Soldier Fly Larvae,” “poultry waste,” “sustainable waste management,” and “circular economy,” combined with Boolean operators (e.g., AND, OR). Inclusion criteria comprised peer-reviewed articles, conference proceedings, and book chapters in English addressing BSFL applications in poultry waste management or related waste-to-resource processes globally. Exclusion criteria included studies focused solely on other waste types or non-BSFL methods. Articles were screened by title and abstract, followed by full-text review to ensure relevance.

## 2 The poultry waste challenge: characterization and environmental footprint

**FIGURE 1:**
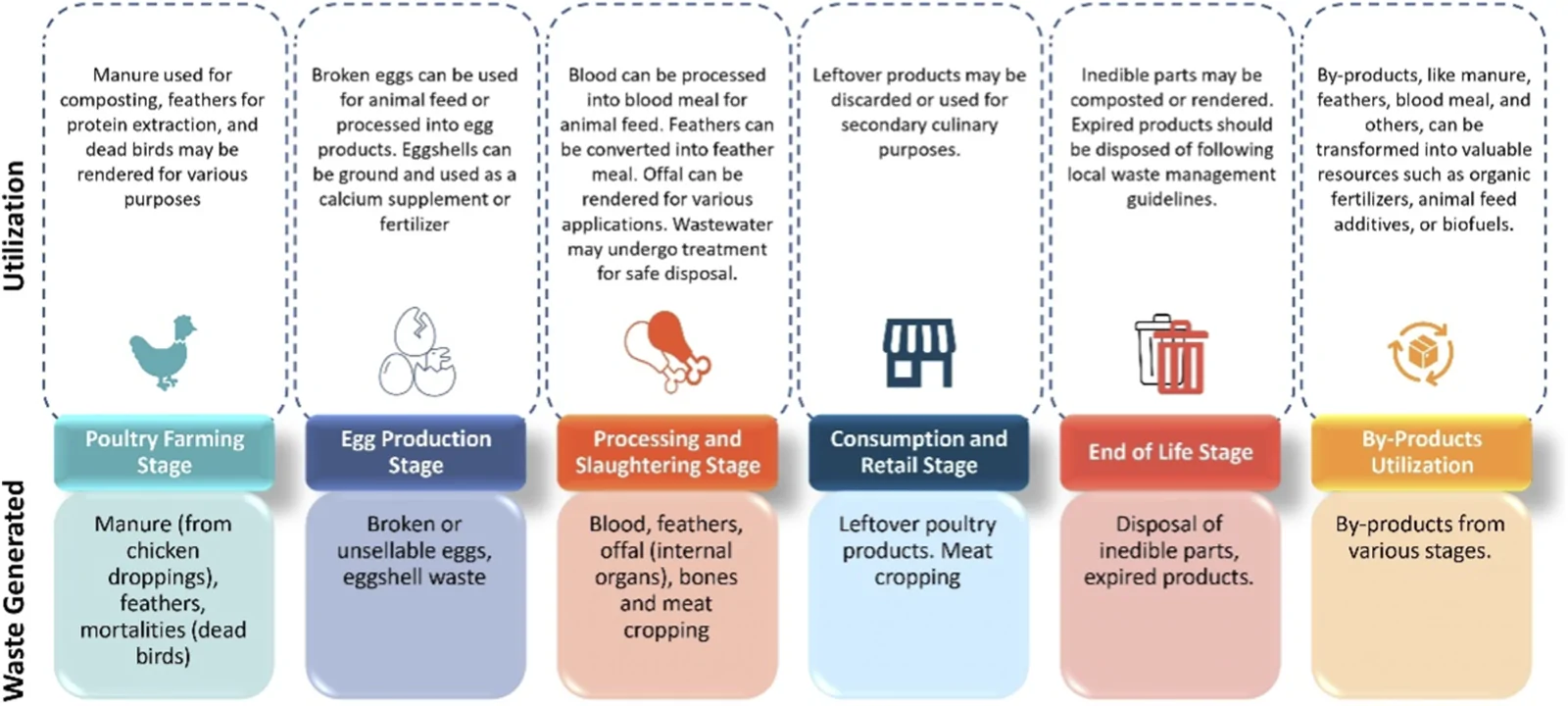
Management of Waste at Various Stages of Poultry Production and Its conventional Application.

Globally, a large amount of various poultry waste generated and to manage this waste at various stages of poultry production there are several conventional methods available as given in Figure 1.

### 2.1 Hatchery by-products

The poultry hatchery generates a significant quantity of both solid and liquid wastes, posing a considerable challenge in its proper disposal. The management and disposal of hatchery waste pose significant challenges for the industry. Its rapid decomposition makes it difficult to store for extended periods, and its high moisture content adds to the cost of transportation. As a result, disposal is often an expensive and strenuous operation (Miller, 1984). Hatchery waste encompasses leftovers in the hatchery trays, once marketable chicks have been separated. This includes discarded hatched chick shells, infertile eggs, nonviable embryos, and deceased chicks (Ilian and Salman, 1986; Glatz et al., 2011). Conventional methods for handling hatchery waste involve options such as landfilling, composting, rendering, and incineration (Ramli et al., 2021). In parallel, wastewater disposal is typically achieved through irrigation or direct discharge into the sewer system (Glatz et al., 2011). To segregate the liquid and solid components of hatchery waste, various techniques are employed. These include separation by spinning, inclined screens, and the use of a flexible multi-layer filter (Glatz et al., 2011). Another system utilizes a conveyor with upper and lower rollers to isolate liquid and solid waste components (Prabakaran and Valavan, 2021). In some instances, a stream of gas is applied to separate lighter materials from heavier ones within hatchery waste (Glatz et al., 2011).

An alternative method employs a moving belt with fixed gaps that allow only chicks to pass through, retaining shells and unhatched eggs on the belt (Shamsuddoha and Woodside, 2022). Additionally, eggshell membrane can serve as an adsorbent for removing reactive dyes from colored waste effluents and eliminating heavy metal ions from dilute waste solutions (Tsai et al., 2006). To further repurpose hatchery waste, eggshells can be composted with other organic materials to enhance the mineral content of the resulting compost. This mixture can then be blended with garden soil for use as a fertilizer (Madhavi and Chakraborty, 2016; Kinaytürk et al., 2021). Eggshells may also find application in construction by enhancing the strength of cement (Chong et al., 2020). Most hatcheries store solid and liquid waste in bins before disposal, with some adopting bio bins to facilitate initial composting. This approach helps mitigate odors and prevents fly and rodent infestation (Glatz et al., 2011). For solid waste treatment, options like composting and anaerobic digestion systems are available (Lin et al., 2018). The converting hatchery waste into animal feed offers a solution to the pollution problem while creating a valuable source of high-quality animal protein (Abiola and Onun Kwor, 2004). Research by Abiola et al. (2012) and Glatz et al. (2011) has highlighted the high protein content and substantial levels of calcium and phosphorus in hatchery waste, making it a promising animal feedstuff.

### 2.2 Abattoir effluents and solid waste

Substantial quantity of waste is generated during meat, egg production and while poultry processing activities. According to UN environment programme report, globally each year, generates total solid waste between 2.1 billion and 2.3 billion tonnes and it may increase to 3.8 billion tonnes by 2050 (UNEP, 2026). Among the total solid waste, slaughterhouse wastes are one of the major group that remain unutilized (Mozhiarasi and Natarajan, 2025). According to one survey, globally the processing waste from poultry slaughterhouse was 45.9 million tons (Seidavi et al., 2019b). The remarkable expansion of the global poultry industry has resulted in the production of millions of tons of chicken waste, encompassing a diverse array of materials, including heads, necks, legs, blood, internal organs, feathers, and inedible carcasses. Throughout the poultry production process, which includes hatcheries, farms, and processing plants, various forms of waste are generated. These encompass solid waste, such as paddy husk, wood shavings, excreta, feathers, eggshells, sludge, and deceased birds, as well as liquid waste, primarily consisting of wash water and cooking losses during rendering.

### 2.3 On-farm residues: litter, manure and mortality birds

The poultry waste generated from poultry industry pose significant environmental and human health risks but as it contains substantial amounts of organic and inorganic nutrients, making them potential candidates for recycling (Chen and Jiang, 2014; Vaishnav et al., 2023). In poultry production, three primary waste streams of concern are deep litter associated with broiler production, cage litter from laying hens, and dead birds (Prabakaran and Valavan, 2021). Deep litter mainly consists of bedding materials like sawdust, wood shavings, straw, and hulls, along with accumulated manure, collectively referred to as poultry litter. Nitrogen and phosphorus are the primary nutrients of concern in poultry litter (Bolan et al., 2010; Ashworth et al., 2020). Nitrogen compounds can be dynamic, with uptake by crops or conversion to gases like ammonia, nitrous oxides, or di-nitrogen (Benbi and Richter, 2024), while phosphorus is relatively immobile but may leach into groundwater or surface waters (Gburek et al., 2015). Poultry manure, in the form of dried cage manure or mixed with litter in deep litter farms, is commonly applied to land near poultry production facilities. Excessive nitrogen and phosphorus levels can lead to surface water degradation, and the presence of metals such as copper and zinc in poultry waste should be considered for sustainable nutrient balance in soils (Kacprzak et al., 2023; Angon et al., 2024). Elevated levels of nitrates in drinking water due to nitrogen pollution can have adverse health effects, and excess nitrogen and phosphorus can contribute to the degradation of surface waters (Nieder et al., 2018). Furthermore, the presence of certain metals like copper and zinc in poultry excreta needs consideration and planning for long-term sustainable nutrient balance in soils (Han et al., 2000). The practice of using lime to cover poultry waste is discouraged due to the risk of groundwater and surface water contamination (Bolan et al., 2010).

The decomposition of poultry manure gives rise to odorous gases containing amines, sulfides, and disulfides, which can pose respiratory health risks to humans. These odorous emissions (Ma et al., 2021; Konkol et al., 2022), along with greenhouse gases implicated in ozone depletion and global warming, are released during the handling and storage of poultry waste. Air quality can also be significantly affected by aerial emissions of pollutants from poultry farms, hatcheries, and processing units. Notably, ammonia emissions into the atmosphere are a significant environmental pollutant associated with poultry production (Bist et al., 2023). During open-air fermentation, hydrogen sulfide and volatile organic compounds can be emitted into the atmosphere as pollutants (Huanhong et al., 2023). Particulate matter, especially fine particles resulting from the conversion of ammonia gas into ammonium salts, presents a heightened risk to human health and is challenging to mitigate through conventional dust barrier approaches (Tom, 2007; Bist et al., 2023).

The rapid growth of the poultry industry has led to the generation of substantial waste streams with significant environmental and health implications (Table 1). Effective waste management and recycling practices are imperative to mitigate these challenges and promote sustainable poultry production while addressing issues related to nitrogen and phosphorus pollution, trace metals, odorous gases, greenhouse gas emissions, and air quality.

## 3 Conventional poultry waste management: strategies and limitations

### 3.1 Rendering

India generates approximately 1.07 million tonnes of poultry waste annually, and rendering is the process that transforms this waste into value-added products such as poultry by-product meal (PBPM) through wet or dry heat-based processes (Jayathilakan et al., 2011; Akporube et al., 2023). Rendering results in a product, that is rich in protein, minerals and vitamins, making it suitable for digestion (Ockerman and Basu, 2024). Despite its numerous advantages, the process still contains several constrains, including significant energy and water requirements, odor and volatile organic compounds emission and waste generation (Costa and Akdeniz, 2019; Zhang et al., 2023). In addition to these limittion, it requires substantial economic investment and poses biosecurity risk, restricting its accessability for small and large scale poultry industries (Jayathilakan et al., 2011; Akporube et al., 2023).

### 3.2 Composting

Composting represents a commonly employed biological process for processing poultry waste by reducing pathogens and weed seeds (Waqas et al., 2023). Various composting systems necessitate land requirements and intricate management (Ayilara et al., 2020; Zhang et al., 2023). It also faces multiple challenges such as odor emission, variable pathogen deactivation, large land and time requirment and inconsistent nutrient levels (Rahman et al., 2022; Bist et al., 2024).

### 3.3 Landfilling

Landfilling is another traditionaly used method for managing poultry waste, yet increasing waste production and limited land resources render this method challenging (Akporube et al., 2023). Landfilling also poses serious risks including nitrate and phosphate leaching, groundwater pollution, and methane release (Anand et al., 2021; Wang et al., 2022). In addition, this method faces operational challenges including rising land and transportation costs, ammonia volatilization, attraction of pests, odor generation, and resistance from communities regarding landfill expansion. Moreover, this method contraindicts the circular economy principles by immobilizing valuable nutrients and energy potential, reinforcing its status as a last-resort disposal option rather than a sustainable management strategy.

### 3.4 Anaerobic digestion

Anaerobic digestion (AD) is a process that transforms waste into biogas *via* sequential microbial processes, providing renewable energy and nutrient-rich digestate (Themelis and Ulloa, 2007). Nonetheless, poultry waste with elevated nitrogen levels frequently leads to ammonia inhibition, which reduces methane production and disrupts the digestion process (Nordahl et al., 2020). It also presents operational complexities such as sensitivity to pH and temperature, hydrogen sulfide generation, and difficulties in managing digestate (Rubežius et al., 2020; Marchioro et al., 2018).

### 3.5 Lipid extraction for biodiesel

The byproducts generated from poultry slaughter, especially chicken fat, can be transformed into biodiesel through esterification and transesterification processes (Ejikeme et al., 2010; Mohiddin et al., 2018). The biodiesel produced through this method complies with quality standards and national biofuel regulations. However, variable lipid content in poultry waste and the need for energy restrict the economic viability (Pikula et al., 2020).

Overall, these conventional poultry waste management methods face drawbacks such as, substantial resource requirement, environmental impact contributes in pollution and green house gas emission which often contraindicates the circular economy concept for waste management (Table 1). Given these constraints, there is a pressing demand for innovative and biologically effective strategies. In this context, bioconversion utilizing Black Soldier Fly Larvae presents a viable option that aids in converting poultry waste into valuable products while improving sustainability and resource recovery (Table 2).

**TABLE 2:** Technological synthesis and comparative performance metrics of black soldier fly larve.

Performance metric	BSFL bioconversion	Conventional composting	Anaerobic digestion	References
GHG Emissions (CO_2_ eq/t)	12–17 kg	341.27 kg	∼225 kg (34% reduction vs. landfill)	Lindberg et al. (2022), Mertenat et al. (2019)
Nutrient Recovery (N)	34.4% (Biomass) + Frass	Low to Moderate	High (80%–90% in Digestate)	Smetana et al. (2019)
Waste Mass Reduction	50%–80%	40%–50%	20%–30%	Amrul et al. (2022)
Processing Time	12–21 days	60–180 days	20–40 days	Lohri et al. (2017)
Land Requirement	50 m^2^/t/day	185–530 m^2^/t/day	High Infrastructure	Maina et al. (2020)
Primary Output	Protein/Lipid/Frass	Soil Conditioner	Biogas/Methane/Digestate	Ungurean and Vlăduț (2026), Tariq et al. (2025)
Globle Economic market	High (USD 422.8 Million)	Moderate (Low CAPEX)	Moderate (Energy Revenue)	Market Research Report (2025), Leipertz et al. (2024)

## 4 Black soldier fly *(Hermetia illucens*): biology and bioconversion potential

### 4.1 The black soldier fly: an overview of a key bioconversion agent

The Black Soldier Fly (BSF), a harmless insect with the potential to solve two of modern agriculture’s growing problems, namely, as an alternative protein source for animal feeds and disposal of organic wastes, and byproducts. The insect was found indigenous to the warm tropical and temperate zones of the American continents (Guilliet et al., 2022). Climate change and human activities facilitated its spread to other continents such as Europe, Asia, and Australia (Kaya et al., 2021). As a result, the BSF was described as native to almost 80% of the world between latitudes 46°N and 42°S (Martínez-Sánchez et al., 2011). Roy et al. (2026) characterized the fly species as a large, black insect with bare eyes composed of uniformly small facets. The face is black, featuring a distinct white median stripe at the front. Furthermore, the authors also noted that this fly species has a cosmopolitan distribution, occurring worldwide.

### 4.2 Ecological niche and optimal rearing parameters

Several studies reported that the BSF was sensitive to abiotic factors of the environment (Richard, 2015). Their requirements varied with the stage of development, and therefore it was opined that to achieve successful breeding in confinement, the conditions should be monitored, regulated and ensured that they conform to the requirements. These include parameters like temperature, relative humidity, light, diet, and pupation substrate. However, Diener et al. (2011a) concluded that *Hermetia illucens* was an extremely resistant species capable of dealing with demanding environmental conditions, such as drought, food shortage, or oxygen deficiency. The economic feasibility of a BSF system depended, among other factors, on the larval biomass produced from a certain amount of waste, in other words, the waste-to-biomass conversion ratio. The ratio varied with the nature of input materials, and might range from as little as 3% for biogas digestate to 23% on a wet-weight basis when working with fresh human excreta (Newton et al., 2005; Lalander et al., 2013; Banks et al., 2014; Spranghers et al., 2017).

### 4.3 Strategic feeding regimes and waste loading rates for maximum throughput

To study the strategic feeding regime, Banks et al. (2014) compared the two feeding regimes, i.e., lumpsum feeding and continuous feeding and observed the growth pattern of black soldier fly larvae (BSFL). In the study they reported significant differences between BSFL groups of different feeding regimes, with maximum throughput observed in larvae fed on lump sum mode as compared to continuous feeding. On contrary to the earlier study, Mutafela (2015) observed where continuous regime fed larvae gained maximum growth. Most research on BSFL rearing even for waste management of the different materials have mostly dwelt with single substrates as feed source (Manyara, 2018). Though, Diener et al. (2011a) had applied BSF technology in the management of mixed municipal markets in Indonesia, a typical waste stream was highly variable, being made of several different components and its characteristic was dependent on the life style of producers (Fowles and Nansen, 2020). Based on the status of people from different countries, the waste generation will differ, so the mixture of the waste is different in devloped and developing countries. Also, the throughput of the BSF technique is more when we uses the mixed substrate as compared to the single substrate (Rehman et al., 2017).

## 5 BSFL-mediated bioconversion of poultry waste: mechanisms and applications

### 5.1 Mechanisms of waste degradation and nutrient upcycling by BSFL

BSFL degrade the waste material through an extensive digestive process which includes biological processes, such as mechanical breakdown through larval feeding, enzymatic digestion (e.g., protease and lipase activity), and microbial symbiosis in the BSFL gut (Tariq et al., 2025). The different part of the digestibve system works in unison, mostly midgut is primary site for the enzymatice digestion with the help of enzymes such as proteases, lipases, and amylases (Ganvir et al., 2022). The mechanical breakdown in BSF is assisted with robust mandibular–maxillary complex that helps in consuming semi liquid waste. Moreover, the larval gut microbiota which mostly contains the Fermicutes and proteobacteria helps in the microbial digestion of complex organic waste materials (Tariq et al., 2025).

Along with the poultry waste, several other waste has been used as a substrate for BSFL mediated bioconversion. In the study by Myers et al. (2008), they reported that the larvae rear on the dairy manure substrate took longer to develop to the prepupal stage; and able to reduce the available P by 61%–70% and N by 30%–50% across treatments. In one more study by Spranghers et al. (2017), they used organic waste substrate, reported that there was no significant correlation between protein and EE contents of the organic waste substrate and those of the prepupae (*R* ^2^ = 0.349; P = 0.409 for protein and *R* ^2^ = 0.054; P = 0.768 for EE). However, a high correlation was observed between the ash contents of substrates and prepupae (*R* ^2^ = 0.954; P = 0.023). And they concluded that since protein content and quality were high and comparable for prepupae reared on different substrates, BSF could be an interesting protein source for animal feeds in comparison with conventional protein source for animal feed as reported by Van Eys et al. (2004). Similarly, several researcher observed that the bioconversion of waste using BSFL will be depend on the composion of the waste material used (Spranghers et al., 2017)

### 5.2 Treatment efficacy across diverse poultry waste streams

Globally, the poultry industry produces a large amount of waste like hatchery waste, slaughterhouse waste and litter waste (Mozhiarasi and Natarajan, 2025). The hatchery waste which includes empty shells, infertile eggs, dead embryos, late hatchings and dead chickens, which having high moisture content nearly about 43%–71%. Considering the nutritive value of the hatchery waste, it contains nearly 33% protein, 29% fat, 12% fibre and less than 5% other essential nutrients. Overall, if we see the total volume of hatchery waste generated is nearly 0.16 kg DM per kg chicks (Abiola and Onun Kwor, 2004; Glatz et al., 2011). Whereas, slaughterhouse waste which contains feathers, offals, blood, *etc.*, having 54% moisture content, also high in protein content. Volume generated from slaughter waste is nearly 35% bird weight (Kazemi-Bonchenari et al., 2017; Mozhiarasi and Natarajan, 2025). Finally, the litter produced in poultry farms like bedding manuar and spilled feed, contains 22%–30% moisture, will have high N, P content which cause water pollution (Tabler and Berry, 2003; Maj, 2022). These diverse waste streams exhibit distinct physicochemical profiles that influence bioconversion potential, as summarized in Table 3, which highlights BSFL treatment efficacy variations across types. In urban areas of developing countries, poultry waste management is one of the very serious environmental problems (Diener et al., 2009; Hoornweg and Bhada-Tata, 2012). Conversion of poultry waste into the useful product can help to solve the problems related to waste disposal. Various value-added products has been produced from the poultry waste, which has created a huge potential in waste management and also impacted on the economic and environmental benefits (Zhang et al., 2023). In the last 2 decades, the management of solid waste has updated from dumping and burning to new technologies that focus on reduction in consumption, reuse, and recycling (Mutafela, 2015).

**TABLE 3:** Comparative performance of BSFL bioconversion across poultry waste types: Key metrics and variations.

Waste type	Moisture content (%)	Key nutrients (% DM)	Bioconversion efficiency (% waste to biomass)	Waste reduction (% DM)	Key conditions/Notes	References
Hatchery Waste (e.g., shells, infertile eggs, dead embryos)	43–71	Protein: 33; Fat: 29; Fiber: 12	31.84 (mixed with farm/slaughterhouse wastes)	51–52	High moisture limits storage; 0.16 kg DM/kg chicks generated; faster degradation but variable yields due to heterogeneity	Niranjan et al. (2024); Abiola and Onun Kwor (2004); Glatz et al. (2011)
Slaughterhouse Waste (e.g., feathers, offal, blood)	54	High protein (unspecified); ∼35% bird weight	7–15 (co-digested with manure)	Not reported	High protein supports biomass but risks pathogen carryover; co-digestion enhances stability but lowers isolated efficiency	Lalander et al. (2019); Kazemi-Bonchenari et al. (2017)
Poultry Litter/Manure (e.g., bedding, excreta, spilled feed)	22–30	High N/P (e.g., N: variable, P: immobile); C/Fe elevated in deep litter	15.31 (chicken manure); up to 28.6% weight gain proxy	52–53	Optimal at 60% moisture, 100 mg feed rate; ammonia inhibition common; higher reduction than hatchery but slower development vs. mixed wastes	Xiao et al. (2020); Mazza et al. (2020); Tabler and Berry (2003); Maj (2022); Diener et al. (2009)

A novel technique to recycle the waste is the use of insects, which degrade the waste with reduction in pathogens and odor (Miranda et al., 2019). The black soldier fly (BSF) is one of the best solutions for poultry waste management as it is capable of digesting large quantities of manure and can be used as an alternative protein source (Miranda et al., 2019). Considering the treatment efficacy of the BSF, few studies have been conducted using poultry waste as a substrate. In the recent study by Niranjan et al. (2024), they used hatcheries, poultry farms, and slaughterhouses waste as a substrate. They found a higher bioconversion efficiency of 31.84% with an effective material reduction of 52.36% (Niranjan et al., 2024). This high efficiency is attributed to optimized environmental conditioning, including a substrate moisture of 60%–80% and a balanced C/N ratio of approximately 37.5, which maximizes larval growth compared to mono-substrates.

In another study, where they used poultry manure and slaughterhouse waste as a substrate, they observed a 7%–15% waste-to-biomass conversion ratio (Lalander et al., 2019). Such variability in bioconversion rates often stems from differences in larval density—typically optimized at 1,000 larvae per unit in high-performing trials—and temperature fluctuations that affect metabolic rates. In one experiment, they used chicken manure and analyzed the transformation of poultry faeces waste by black soldier fly larvae, finding that the conversion rate of chicken manure was 15.31% (Xiao et al., 2020). Similarly, in the study done by Mazza et al. (2020), BSF fed on chicken manure resulted in 28.6% more weight gain and a higher manure reduction rate (52.91%) (Mazza et al., 2020). Hence, BSF has a great treatment efficacy for poultry waste and can be used as a protein alternate source for livestock.

### 5.3 Valorization of organic matter: biomass generation and nutrient transformation

Bioconversion was the process of transferring nutrients in the organics into the body components of a living organism that consumed the waste, such as earthworms, insect larvae, or microbes. The process resulted useful products such as a nutritious biomass and fertilizer (Barry, 2004). However, many species of flies and earthworms could not process a wide range of organic wastes and required specific nutrition to be good and effective bioconverters (Latsamy and Preston, 2008; Morales and Wolff, 2010; Zhang et al., 2012). In addition, some flies and microbes were human pests and disease vectors (Sasaki et al., 2000; De Jesús et al., 2004). This was unlike BSF. which was not known as a pest, pathogen vector, or nuisance of any kind to humans or the animals (Erickson et al., 2004). BSF bioconversion technique involves the artificial growing of BSF larvae on a suitable substrate medium to facilitate the hatching of eggs to larvae. The reared larvae grew on the waste feedstock from which they extracted nutrients and reduce the waste mass. The larval polyphagous nature and robust digestive system enabled them to feed on a wide range of decaying organic materials both of animal and plant origin whereas the voracious appetite facilitates the consumption of large amounts of organic waste during their growth cycle (Mutafela, 2015). In this regard, bioconversion potential of BSF larvae was described as one of the most promising and sustainable methods of managing organic wastes (Zhou et al., 2013). At the end of the process, larvae are harvested and may be post-processed into a suitable animal feed product. The waste residue can also be further processed and potentially sold or used as soil amendment with fertilizing properties. The antibacterial and antifungal activity of BSF larvae made it safe for use in agricultural farming (Humphrey, 2009; Banks et al., 2014; Nguyen et al., 2015). In this way, a BSF technology not only looked into organic waste management but also a value addition process that enabled even environmentally hazardous wastes which become a source of income generation and employment creation (Mutafela, 2015).

## 6 Optimizing BSFL rearing systems for efficient poultry waste valorization

### 6.1 Substrate formulation and pre-treatment strategies for enhanced performance

Substrate usage and suitability Major BSFL growth parameters such as development time, feed conversion efficiency, mortality, pupae weight and nutrient composition were strongly affected by the growth substrate (Zheng et al., 2013). Therefore, the commercial scale application of the technology would demand usage of substrates that could yield good quality larvae within a short duration and reduce losses through mortality is a necessary. Organic waste materials were also highly heterogeneous in nature and variable in terms of moisture and nutrient content and therefore generalized applications of findings was almost impossible (Holmes et al., 2012). The substrate formulation mostly done by mixing various organic wastes to optimize the nutrient content of the substrate (Siddiqui et al., 2024). Whereas, the pretreatment is necessary for enhancing the performance of larva. There are various strategies involved such as physical, chemical or biological (Isibika, 2022). In the physical method, grinding can be done which helps in increasing the surface area and subsequently the digestion of the substrate. The chemical method uses the adding of other chemical which helps in the braking down the complex substrate. Whereas, the biological methods can increase the enzyme activity with the help of microorganisms like Trichoderma spp. and Bacillis subtilis (Siddiqui et al., 2024).

Manyara (2018) reported that among the four substrates used in his study food residue produced the heaviest prepupa and highest yield followed by brewer waste with fecal sludge in third place. Nguyen et al. (2015) compared development rate, size and mortality of BSF larvae fed on poultry, feed, pig liver, pig manure, fish renderings, and kitchen food waste and reported that larvae fed on kitchen food waste had the fastest growth, heaviest biomass and yields were attributed to higher calorie content.

### 6.2 Factors governing larval growth and survival

Successful rearing of black soldier fly larvae (BSFL) for sustainable poultry waste management relies on optimizing dietary substrates and environmental conditions to enhance growth and survival, which is crucial for circular economy applications. The nutrient composition of the substrate, particularly protein and volatile solids, is pivotal for BSFL development. While BSFL can survive on low-protein substrates, their growth is significantly limited without adequate protein, with optimal larval weight requiring high protein and minimal fat content (Broeckx et al., 2021). Additionally, the moisture content and total volatile solids in the substrate influence bioconversion efficiency and development time (Lalander et al., 2019). The quality of poultry waste directly affects performance metrics such as the feed conversion ratio, emphasizing the need for balanced diets rather than merely protein-rich ones (Niranjan et al., 2024). Beyond diet, the gut microbiome plays a crucial role in enhancing nutrient utilization, with supplements like *Bacillus subtilis* or yeast shown to boost growth and feed conversion (Seyedalmoosavi et al., 2022). However, factors such as high zinc levels, anaerobic conditions, or stagnating liquids can negatively impact larval yield (Diener et al., 2009). Furthermore, environmental conditions, especially temperature, significantly affect the BSFL life cycle, which can range from 41 to 131 days depending on ambient conditions (Seyedalmoosavi et al., 2022). This underscores the importance of managing temperature alongside diet to maximize efficiency. In summary, effective BSFL rearing for poultry waste valorization requires precise management of substrate nutrition, the gut microbiome, and temperature to optimize growth and survival, aligning with sustainable waste management goals.

### 6.3 Rearing system design: environmental control and process optimization

Optimization of black soldier fly larvae production on organic waste for BSFL to serve effectively in the dual roles of biomass production and attendant waste reduction, establishment of a production system with a feeding strategy that specified important substrate factors that affect BSFL feeding behavior, growth and development is necessary (Devic and Maquart, 2015). These factors included aspects such as larvae feeding amounts (feed rate), feeding frequency (regime), substrate types and substrate combination ratios, substrate depth, larvae stocking densities, substrate moisture content, environmental rearing conditions such as temperature and relative humidity; and size of substrate particles among others (Holmes et al., 2012).

According to Diener et al. (2009), the feed consumption rate of BSF larvae depend on the size of larva and the type of feed. The study subsequently predicted that a feeding rate of 100 mg of chicken feed at 60% moisture as the optimal tradeoff feed rate between nutrient rich prepupa and high waste consumption in the shortest time. The effective rearing of the larvae needs well designed system to accommodate the different stages of BSF. Small scale rearing is suitable for the household or small-scale farmers which often used simple vessel or bins with depths of 20–60 cm having drainage holes (Morales-Ramos et al., 2024). These systems incorporate egg-laying surfaces, such as stacked corrugated cardboard, positioned above moist substrates to attract ovipositing females. Whereas, in case of large-scale rearing of BSF needs large room with individual bays for different waste sources, optimizing space and facilitating waste segregation (Sankara et al., 2023). In case of adult flies, requires lumite screens to prevent pest, also provision for water, vegetation and sugar (Sankara et al., 2023). Rearing system completely depends on the environmental conditions which are very critical for growth and development of BSF. The optimum temperature of approximately 27 °C needed for egg viability peaking at 30 °C, whereas, relative humidity should be in between 50% and 70%. The moisture percentage at the system is very important, with ensuring the proper drainage to prevent waterlogging and foul odors (Singh et al., 2022). However, the light intensity of 63 μmol m^-2^ s^-1^ is required for mating in adult flies (Seyedalmoosavi et al., 2022).

Optimization of the rearing system is also depending on the substrate selection, feeding regimes, and harvesting method. Poultry waste, particularly manure, is an excellent substrate due to its high protein content, which supports rapid larval growth and high biomass yields (Rehman et al., 2023). Several studies suggested that combination of poultry waste with vegetable waste enhances the nutritional content and biomass of larvae (Rehman et al., 2023). Overall, the closed rearing system containing the optimum environmental conditions and substrate provides the optimum condition for proper growth and development of BSF larvae.

## 7 Enzymatic, microbial, and molecular dynamics of black soldier fly larvae–mediated poultry waste bioconversion

BSFL systems has potensial to reduce large waste volume, whereas, its outcomes are solely controlled by the enzymatic, microbial and molecular dynamics of BSFL.

### 7.1 Genomic foundations and molecular adaptations for waste processing

The remarkable bioconversion ability of *H. illucens* is inherently baed on its unique genomic architecture, which has evolved to facilitate survival and growth in nutrient-rich, microbe abundant settings (Tariq et al., 2025). The genome of the black soldier fly (Hermetia illucens), a key species for sustainable bioconversion, is relatively large compared to other dipterans, with a size estimated at approximately 1.01–1.10 Gb (Kaya et al., 2021). Chromosome-level assemblies have revealed a karyotype of 2n = 14, consisting of six autosomes and one pair of sex chromosomes (X/Y) (Kaya et al., 2021). The genome is highly repetitive, with nearly 65%–67% comprised of transposable elements and repetitive sequences (Kaya et al., 2021). Functional annotation has identified between 16,478 and 16,770 protein-coding genes, with a notable expansion in gene families related to digestion (e.g., amylases, lysozymes) and immunity (e.g., antimicrobial peptides), which likely underpins the larvae’s exceptional ability to thrive on diverse and decaying organic substrates (Kaya et al., 2021). This genetic intricacy supports the larvae’s broad range of enzymes and immune functions, conferring notable metabolic flexibility (Tariq et al., 2025). Notably, expansions in specific gene families directly aid in detoxification and nutrient absorption processes.

Among these, the Cytochrome P450 (CYP450) gene family is markedly expanded, enhancing the larvae’s capacity to metabolize xenobiotics, organic pollutants, and heavy metals often found in poultry manure and abattoir effluents (Tariq et al., 2025). Silultaneously, the genomic expansion of olfactory receptors and odorant-binding proteins (OBPs) allows BSFL to sense volatile organic compounds released during the breakdown of organic matter, directing larvae toward the best feeding sources (Tariq et al., 2025). Improvements in molecular breeding illustrate the promise for focused optimization of these characteristics; CRISPR/Cas9-based modification of growth regulators such as the *giant (gt)* gene has led to larvae showing a bioconversion efficiency increase of up to 13.1% while also boosting reproductive ability, underscoring the viability of enhancing detoxification, immunity, and chemoreception processes at the molecular level (Wang et al., 2025). However, the implementation of these modified strains in practical waste valorization systems faces significant regulatory hurdles. In many regions, the use of genetically modified organisms (GMOs) in the production of animal feed is subject to rigorous safety assessments and ‘precautionary principle’ protocols, which currently limit the transition of CRISPR-enhanced BSFL from laboratory settings to industrial-scale feed production.

In addition to metabolic genes, BSFL have a wide array of immune-associated genes, featuring as many as 50 potential antimicrobial peptides (AMPs) like defensins, cecropins, and attacins (Tariq et al., 2025). These AMPs serve as a crucial barrier against the significant pathogen levels often linked to poultry waste substrates (Camperio et al., 2025). Transcriptomic studies reveal that AMP expression is highly responsive; contact with Gram-negative bacteria triggers peptidoglycan recognition proteins (PGRPs) and related signaling molecules like Relish, leading to swift AMP release that maintains gut microbial balance (Shah et al., 2024).

### 7.2 Digestive architecture as a molecular bio-reactor

The digestive system of BSFL functions as a highly specialized molecular bio-reactor marked by significant regional differentiation and steep physicochemical gradients (Zheng et al., 2025). The midgut is divided into anterior, middle, and posterior parts, each having unique pH levels and enzyme profiles that regulate the stepwise degradation of poultry waste components.

The anterior midgut has a mildly acidic pH of around 6, aiding in early hydrolysis and lubrication, whereas the middle midgut presents a highly acidic pH of about 2, acting as a molecular ‘sterilization zone’ that deactivates consumed pathogens like *Salmonella enterica* and *Escherichia coli*, as well as enhances acid hydrolysis of complex proteins and solubilization of inorganic minerals (Niranjan et al., 2024; Zheng et al., 2025). This is succeeded by a distinctly alkaline posterior midgut (pH ∼8.5), tailored for alkaline enzymatic digestion and nutrient uptake (Zheng et al., 2025). The salivary glands and esophagus also aid in initial processing by releasing mucoproteins that improve lubrication and mechanical breakdown through a robust muscular layer (Zheng et al., 2025).

BSFL demonstrate significant enzymatic flexibility, enabling their digestive system to adapt in real time to the nutrient concentration of the substrate (Bruno et al., 2024). Transcriptomic analyses indicate that larvae grown on low-nutrient materials like poultry litter or the organic portion of municipal solid waste increase the expression of genes for proteases, amylases, and lipases. The enzymatic composition of the gut is primarily characterized by trypsin-like proteases, leucine arylamidase, and various α- and β-glycosidases, facilitating the effective transformation of nutrient-deficient poultry waste into larval biomass that is rich in proteins and lipids (Zheng et al., 2025).

### 7.3 Enzymatic and microbial synergy in nutrient upcycling

The bioconversion of poultry waste by BSFL is a collaborative process that includes the larval host and its associated gut microbiota (Shao et al., 2023). The gut microbiome consists of a dynamic community primarily made up of Firmicutes, Bacteroidetes, Proteobacteria, and Actinobacteriota, featuring taxa like *Bacillus subtilis*, *Dysgonomonas*, *Pseudomonas*, *Enterococcus*, and *Bacillus cereus* (Shao et al., 2023; Yu et al., 2023; Memon et al., 2025). These microorganisms generate external enzymes, such as cellulases, α-amylases, and lipases, that support host-sourced enzymes and enhance the decomposition of intricate organic structures.

The characteristics of substrates have a significant impact on microbial community composition. Diets that include poultry manure enhance the abundance of genera like *Lactobacillus* and *Clostridium*, aiding in the breakdown of residual carbohydrates and proteins (Shao et al., 2023). Host-mediated filtering further modifies the microbiota into a cohesive yet highly functional community, selectively enhancing taxa like *Lysinibacillus* and *Cytobacillus*, which have extensive enzymatic capabilities and support gut homeostasis (Memon et al., 2025).

The significance of this host–microbe synergy is evident from research conducting comparisons between germ-free (GF) and gnotobiotic (GB) larvae. In protein-rich substrates mimicking abattoir waste, GB larvae reached a 73.44% greater protein reduction rate than GF larvae, with this variance directly associated with increased activity of gut proteinases like trypsin and peptidase, stimulated by microbial taxa such as *Pseudomonas* spp. and *Issatchenkia* spp. (Yu et al., 2023). These results highlight the crucial function of the gut microbiome as a vital catalyst for waste conversion.

### 7.4 Molecular mechanisms of recalcitrant poultry waste degradation

Feathers, a significant component of poultry waste, consist mainly of β-keratin, a resilient fibrous protein reinforced by dense β-pleated sheets and substantial disulfide cross-linking, making it impervious to traditional proteolytic enzymes like pepsin (Li, 2019). The BSFL system typically supported by specific external microbes utilizes a synchronized three-step molecular process, sulfitolysis, proteolysis, and deamination, to transform feather waste (Gupta and Singh, 2014).

Disulfide reductases (EC 1.8.1.8) begin sulfitolysis by breaking disulfide bonds, which weakens the keratin matrix. Next, proteolysis is mainly carried out by subtilisin-like serine proteases (S8 family), which hydrolyzes keratin into short peptides and free amino acids (Almahasheer et al., 2022). Isolates of *Bacillus subtilis* and *Bacillus cereus* from poultry environments can enhance keratinase activity by as much as 50 times under optimal conditions (pH ∼8.5; 37 °C–55 °C), liberating essential amino acids like methionine, cysteine, and leucine (Almahasheer et al., 2022). At the molecular scale, the uptake of approximately 10 kDa keratin monomers promotes additional expression of keratinolytic genes, creating a positive feedback mechanism that guarantees complete substrate hydrolysis (Cao et al., 2024).

In addition, other by-products such as deep litter have considerable lignocellulosic content originating from bedding materials such as straw or wood shavings (Maurya and Singh, 2024; Niranjan et al., 2024). Associated microbiota with BSFL can address this fraction by producing Carbohydrate-Active Enzymes (CAZymes) (Memon et al., 2025). Metatranscriptomic investigations show a prevalence of glycoside hydrolase families, especially GH51 and GH43_16, arranged in polysaccharide utilization loci that break down lignin and cellulose into fermentable sugars, which are then transformed into larval lipid reserves (Kariuki et al., 2023). Synergistic interactions with thermophilic cellulose degraders, such as *Bacillus mojavensis* improve lignocellulose breakdown by more than 68%, showcasing the efficacy of combined biological pretreatment methods (Shao et al., 2023).

### 7.5 Molecular-scale nitrogen and phosphorus recycling

The transformation of nitrogen and phosphorus is a key aspect of circular nutrient recovery facilitated by BSFL (Kullan et al., 2024). BSFL bioconversion efficiently transfers nitrogen from poultry waste into larval biomass, decreasing ammonia emissions by as much as 25% in comparison to composting (Chen et al., 2025). Nitrogen recovery efficiencies vary between 34.9% and 83.0%, influenced by substrate C:N ratios (Froonickx et al., 2023). Metagenomic studies of frass-amended soils show an increase in carbon fixation genes (pycA, pycB) and denitrification genes (nirB, nirK, nirS), suggesting improved nitrogen stabilization in soil ecosystems (Zhao et al., 2025).

In poultry manure, phosphorus is often found in immobilized organic forms such as phytic acid (Pan and Cai, 2023). BSFL and related phosphate-solubilizing bacteria, such as *Pseudomonas* and *Nocardiopsis*, enhance phosphorus activation by releasing phytases and phosphatases, as well as generating organic acids (e.g., citric and malic acids) that solubilize inorganic phosphorus through proton exchange (Pan and Cai, 2023; Reyer et al., 2025). This method improves the phosphorus available to plants in the finished compost by 24%–29% (Chen et al., 2023). Simultaneously, metal homeostasis processes involving genes like *pcoC*, *zurR*, and *zntB* modulate Cu, Zn, and various heavy metals, improving the agronomic safety of residues derived from BSFL (Bohm et al., 2022).

### 7.6 Multi-omics insights into substrate adaptation and stress responses

The molecular adaptability of BSFL is further emphasized by transcriptomic and metabolomic changes in reaction to various waste substrates (Zhang et al., 2024). Larvae nurtured on nutrient-dense abattoir byproducts show increased expression of genes related to glycolysis and the tricarboxylic acid cycle, whereas poultry or pig manure trigger significant stress responses characterized by upregulation of antioxidant defense mechanisms and short-chain fatty acid metabolism (Zhang et al., 2024).

Long-term adaptation to region-specific poultry waste requires the restructuring of regulatory networks, including the activation of the TOR nutrient-sensing pathway and transcription factor families like zf-C2H2, which adjust metabolic reserves based on substrate composition (Zhang et al., 2025). Furthermore, molecular studies of the Malpighian tubules have revealed that nutrient amino acid transporters from the SLC6 and SLC7 families play crucial roles in nutrient distribution. Silencing transporters like HiNATt raises overall amino acid levels by 77.3%, offering a molecular method for customizing the nutritional composition of BSFL-derived larval meal (Liu et al., 2024).

## 8 Advancing circular economy principles *via* BSFL bioconversion

### 8.1 Economic viability

The global poultry market is poised for robust expansion, with its valuation projected to rise from $384.95 billion in 2024 to $410.98 billion by 2025, reflecting a significant compound annual growth rate (CAGR) of 6.8% (Market Research Report. 2025), necessitate efficient and economically viable management strategies. Traditional methods often present significant drawbacks, in contrast, BSFL farming emerges as a promising alternative, leveraging a circular economy approach by valorizing poultry waste into high-protein larval biomass for animal feed and nutrient-rich frass for organic fertilize, giving economical benefits % (Tambeayuk et al., 2023; Niranjan et al., 2024; Siddiqui et al., 2024).

One primary economic benefit is the reduction in feed costs. Research suggests that BSFL meal inclusion in poultry diets can improve cost-benefit ratios, with a study reporting a CBR of 2.12 for diets with 20% BSFL meal, indicating $2.12 return per dollar invested, compared to lower returns with conventional feeds (Waithaka et al., 2022). A case study demonstrated a 10% reduction in feed expenses for free-range chickens by incorporating BSFL-processed manure, crucial as feed costs constitute roughly 70% of production costs (Chia et al., 2019, which found reduced feed costs and improved economic performance in broiler and pig diets, respectively. Beyond feed, the frass byproduct holds considerable economic value as a high-quality organic fertilizer, meeting increasing demand in sustainable agriculture (Chepkorir et al., 2024).

BSFL farming’s cost-effectiveness is further enhanced by its efficiency and lower environmental impact, translating to reduced compliance costs. This reduction can lower carbon taxes or regulatory penalties, a significant cost for conventional methods. Additionally, BSFL requires less space and achieves faster waste reduction than composting, potentially making it more cost-effective for large-scale operations with limited land (Amrul et al., 2022).

On-farm BSFL treatment units can further reduce costs associated with collection, transportation, and off-site disposal, which can constitute a substantial portion of waste management expenses (Rindhe et al., 2019). By processing waste directly, producers achieve self-sufficiency, contrasting with landfilling’s high transportation and landfill fees. The use of poultry waste as a substrate can turn disposal costs into economic benefits, as waste with a negative value (disposal cost) becomes a resource, potentially lowering overall expenses (Leipertz et al., 2024). However, challenges exist, such as high initial capital investment for BSFL facilities and ensuring a consistent supply of uniform-quality poultry waste, which can affect economic feasibility (Siva Raman et al., 2022). Ongoing research aims to optimize production systems, reduce operational costs, and explore high-value byproducts like chitin and lipids, enhancing competitiveness (Chepkorir et al., 2024). Long-term viability may involve using negative value substrates or targeting niche markets, such as higher welfare production systems with low BSFL inclusion, to manage costs (Leipertz et al., 2024). For instance, skipping processing for alive grown larvae reduces cost increases to 42.2% compared to 53.7% for processed inclusion, suggesting cost-saving strategies (Leipertz et al., 2024).

### 8.2 Pathogen reduction and biosecurity functions of black soldier fly larvae

Adult black soldier flies (BSF) are not known to transmit pathogens to humans; instead, their larval stage plays a critical role in suppressing pathogen transmission and improving biosecurity. Black soldier fly larvae (BSFL) effectively control housefly populations on colonized substrates by preventing oviposition of houseflies, primarily through the secretion of distinctive odor-based infochemicals that repel houseflies and other pest flies (Erickson et al., 2004; Manyara, 2018). This competitive exclusion enables BSFL to outcompete harmful flies for resource utilization while simultaneously reducing microbial loads. BSFL directly contribute to pathogen reduction by consuming microorganisms and through the production of antibacterial and fungicidal compounds, partly mediated by gut-associated bacteria such as *Lysobacter* (Erickson et al., 2004; Zheng et al., 2013). Lalander et al. (2013) demonstrated a significant reduction of *Salmonella* spp. in faecal substrates treated with BSFL, suggesting effective removal of zoonotic *Enterobacteriaceae*. This reduction substantially decreases the risk of disease transmission to animals and humans when BSFL-treated residues are reused as agricultural fertilizers.

As discussed, BSFL maintain biosecurity by reducing pathogen load and antibiotic resistance genes (ARGs) in poultry waste (Tariq et al., 2025). Several studies reported that BSFL has ability to reduce ARGs by more than 99% for several pathogens. Molecular screening studies have depicted the ability of BSFL to reduce ARGs in biosolids by more than 99% for certain pathogens. This enhanced biosecurity effect is largely due to the extreme pH conditions within the larval midgut and the secretion of antimicrobial peptides (AMPs) (Camperio et al., 2025). Key AMPs produced by BSFL, including cecropins and defensins, exert broad-spectrum antimicrobial activity by disrupting bacterial cell membranes or interfering with intracellular targets. These peptides are effective against both Gram-positive bacteria such as *Staphylococcus aureus* and Gram-negative pathogens including *Salmonella* spp. (Camperio et al., 2025). Transcriptomic analyses revealed that larvae downregulate genes associated with metabolic functions (e.g., trypsin and chymotrypsin) to redirect the energy toward immune defense mechanisms. This shift is marked by the upregulation of immune signaling pathways such as PGRP-SA and Relish, emphasizing the dynamic immune adaptability of BSFL (Shah et al., 2024).

In addition to their biosecurity advantages, BSFL have been extensively evaluated as a sustainable feed ingredient. Feeding trials involving cockerels (Hale, 1973), pigs (Newton et al., 1977), and aquaculture species such as catfish and tilapia (Talamuk, 2016) demonstrated that BSF larval meal can successfully replace conventional protein and fat sources such as soybean meal and fish meal. While separation of protein and fat fractions would allow more precise feed formulation, the overall nutritional value of BSFL remains highly favorable. Furthermore, the chitin-rich exoskeleton of prepupal, pupal, and adult stages has been associated with immune-enhancing effects in animals, potentially contributing to improved disease resistance (Lee et al., 2008).

### 8.3 Environmental benefits

It was reported that larvae of the BSF could reduce dry matter in waste by 50% (Sheppard et al., 2002) and house fly, *Musca domestica L.* (*Diptera: Muscidae*) populations by 94%–100% (Miranda et al., 2019). BSF larvae feeding activity reduced the amount of organic waste dumped in open streets and water points by at least 50%–60% (Diener et al., 2011a), resident nutrients such as nitrogen content by 71%, phosphorus and potassium by 52% each, amount of greenhouse gases that was generated from the waste through anaerobic respiration such as CO2, SO2, methane, ammonia and other noxious gases (Van Huis, 2013). The larvae also treated organic leachates that polluted marine and terrestrial environments (Popa and Green, 2012), and clean up oil and grease pollutions that might cause aquatic suffocations by feeding on the spilled oil (Zheng et al., 2013; Banks et al., 2014). Soldier fly prepupae could be easily self-harvested by directing their search for pupation sites into collection bins (Sheppard et al., 2002).

### 8.4 Resource recovery

The oil of BSF could be extracted and used as biodiesel, which can reduce dependence on unsustainable sources such as jatropha plants (Jung et al., 2022). Different types of organic wastes were tried for artificial farming of BSF larvae in confinement. These include livestock manures from large, confined animal feeding operations; palm kernel waste, pig liver, kitchen waste, rendered fish and human fecal waste were summarized in Table 4. Though the larvae were able to transform the waste into a more valuable, more nutritive and less harmful biomass rich in protein (44.4% of DM), lipids (23% of DM), ash (11%–28% of DM and other valuable nutrients such as calcium (5%–8% DM) and phosphorus (Newton et al., 1977; St-Hilaire et al., 2007; Van Huis, 2013).The BSFL biomass composition is favorably compared with that of fishmeal and soya, which contributed towards the supply of over 90% of the protein requirements of animal feeds (Guo et al., 2009). This indicates that BSFL biomass rearing on selected organic wastes could suitably serve as an animal protein source.

**TABLE 4:** Recent research on Black Soldier Fly Larvae (BSFL) applications, summarizing their effectiveness in waste valorization, sustainable feed production, biosecurity, and as a fertilizer, across diverse organic substrates and geographical locations.

Purpose of the study	Treatment/Substrate used	Location	Significant results	Remarks	References
Waste Valorization	Various farm waste, slaughter waste and hatchery waste	India	BSFL effectively reduced poultry waste by 52.36%, demonstrating 31.84% bioconversion efficiency and a 1.25 FCR (P < 0.05). This yielded protein-rich pre-pupae biomass and valuable, *Salmonella*-free residue with a high manure value (C/N ratio 37.5). The process also significantly reduced waste recycling time to 24 days	The novel method successfully transforms poultry waste into commercially valuable byproducts, addressing a critical environmental concern while producing safe, useful resources	Niranjan et al. (2024)
Bio-waste treatment/Waste Valorization	Mill by-products, human faeces, poultry slaughterhouse waste, cow manure, and canteen and vegetable canteen waste	Switzerland	BSFL can convert waste into animal feed, fertilizer, and bioenergy. Pretreatment methods may improve digestibility of substrates with high lignin and cellulose	the formulation of mixed biowaste offers a promising systematic approach for the more efficient and predictable operation of black soldier fly larvae (BSFL) treatment facilities using a range of biowastes	Gold et al. (2020)
Waste Valorization	Food waste; Starter feeds: chick mash (CM), rice bran (RB), and wheat bran (WB)	Kenya	Chick mash as starter feed resulted in highest larval weight, substrate reduction (82.8%), and bioconversion rate (13.8%). Rice bran not recommended as starter feed due to high ash content	Food waste is a suitable substrate for BSFL. BSFL can lower carbon footprint and assist in recycling refuse	Okeyo et al. (2024)
Waste Valorization, Feed Production, Other (Circular Economy)	Fruit and vegetable waste from retail market	Tanzania	Free-range open system produced 19.15 kg of BSFL with 44.34% protein, 20.6% crude fat, and zero heavy metals	Community-led open BSFL system can generate valuable products with minimal resources, fostering circular economy	Chineme et al. (2023)
Waste Valorization, Feed Production	Sludge and Chicken feed (various ratios)	Jordan	100% chicken feed led to highest larval growth and bioconversion rate. 100% sludge achieved highest waste reduction rate (86.2%)	Substrate composition is crucial for optimizing BSFL growth and waste reduction efficiency	Albalawneh et al. (2024)
Waste Valorization, Feed Production, Microbiological Control	Organic wet fraction of municipal household waste (OWF), kitchen/fast food waste (FFW), mushroom feet stems (MF), pig manure slurry, pig manure solids (PMS), secondary sludge	Europe	Larval yields highest on control (chicken feed + water) and FFW. Cadmium bioaccumulated in larvae. Some pesticides and veterinary drugs found in substrates, low concentrations in larvae	Most tested substrates are suitable for rearing BSFL, but Cd monitoring and pathogen control needed	Hoek-Van den Hil et al. (2023)
Waste Valorization	Maize straw, rice straw and wheat straw	China	After bioconversion by BSFL, the residual maize straw was significantly changed in crude protein, crude fat and nitrogen free extract content. By comparing with organic fertilizer, maize straw residue had a potential application in agriculture and nursery as a novel fertilizer	This new alternative method will benefit the comprehensive bioconversion of maize straw with pretreatment by insect	Gao et al. (2019)
Waste Valorization	human faeces, food waste	India	The protein content in the BSFL was not impacted significantly by the quality of the waste stream, whereas the fat content varied substantially. The abiotic factors also impacted the protein and fat content of BSFL.	The investigation led to the estimation of the decomposition rates over a wide range of temperature and relative humidity conditions, which could be useful for the design of large-scale BSFL-based treatment plants	Purkayastha and Sarkar (2023)
Waste Valorization	Wuzhishan pig manure	China	BSFL in growing pig stage showed highest conversion efficiency of pig manure. Synergistic co-cultivation with *Bacillus cereus* and *Bacillus subtilis* enhanced conversion	Optimal BSFL stage and synergistic bacteria significantly improve organic waste conversion	Shao et al. (2023)
Waste Valorization, Microbiological Control	Food waste	Sweden	Pathogen inactivation observed in small-scale BSFL conversion is also applicable to large-scale systems. Larval presence and activity linked to inactivation	Increased water evaporation in larger systems led to higher electrical conductivity in frass. Frass recirculation improved larval yield	Lalander et al. (2025)
Feed Production	Commercial shrimp feed, Fresh BSFL	China	Replacing 25% commercial feed with fresh BSFL increased serum immune enzyme activity, improved water quality (reduced TAN, nitrate, increased pH), and enhanced intestinal microbiota richness and diversity in Pacific white shrimp	Proper replacement of commercial feed with fresh BSFL has positive effects on shrimp immunity, water quality, and gut health	He et al. (2022)
	Soybean meal, Black soldier fly larvae meal (BSF)	Netherlands	Complete replacement of soybean meal with 10% BSF in laying hen diet improved feed efficiency, maintained production performance and egg quality, and increased egg yolk color	Replacing soybean meal with up to 10% BSF in laying hen diet has no adverse effects and can improve feed efficiency	Veldkamp et al. (2024)
	Fish meal (FM), Fish oil (FO), Black soldier fly full-fat larvae meal (BSFL)	Russia	BSFL incorporation (10% and 15%) in Siberian sturgeon diet was more profitable than FM and FO, improved feed efficiency, lowered FM/FO usage, and positively affected gastrointestinal tract development	BSFL can be a more profitable and sustainable alternative to FM and FO in Siberian sturgeon farming	Rawski et al. (2021)
Biosecurity	Fruit and vegetable market waste, farm waste, food industry waste, Rapeseed cake	Nepal	BSFL survival high on food industry waste, lower on high-moisture vegetable waste. Rapeseed cake supplementation improved growth, survival, bioconversion, and larval protein content	BSFL can effectively recycle diverse local organic wastes in developing countries like Nepal, providing a sustainable protein source. Further research on safety needed	Gautam et al. (2025)
	Chicken manure (CM), Kitchen waste (KW)	Kenya	*Providencia* sp. was the most dominant bacterial species in guts of BSFL reared on both CM and KW. Presence of clinically pathogenic strains highlights biosafety risk	Diversity of bacterial genera was higher in fresh rearing substrates compared to BSFL gut and frass. Appropriate pre- and postharvest measures are needed	Shumo et al. (2021)
Fertilizer	Black soldier fly frass fertilizer (BSFFF), Commercial mineral fertilizer, Organic fertilizer (SAFI), Nematicide	Kenya	BSFFF +5% chitin significantly increased potato growth, tuber number and yield, and reduced potato cyst nematode reproduction compared to other treatments	Chitin-fortified BSFFF is a promising and sustainable alternative to commercial fertilizers and nematicides for potato production	Anedo et al. (2025)
	Composted black soldier fly frass fertilizer (BSFFF), Composted brewer’s spent grain (BSG), Commercial organic fertilizer (Evergrow), Mineral (NPK) fertilizer	Kenya	Combination of BSFFF and NPK significantly increased yields of tomatoes, kales, and French beans compared to control and sole NPK. Highest agronomic N use efficiency with sole BSFFF.	Integration of BSFFF and NPK improves soil health, boosts yield, and nutritional quality of vegetable crops in Sub-Saharan Africa	Anyega et al. (2021)
	Black Soldier Fly larvae (BSFL) frass and larval sheddings, Wood chips, Food waste, Livestock manure	Unknown	Inclusion rates of 25%–30% of BSFL frass and sheddings produced compost with generally favorable characteristics (pH, C:N ratio). Phosphorus levels were high	BSFL frass and sheddings can be valuable ingredients in compost production, contributing to nutrient recycling	Kenchanna et al. (2024)
	Black soldier fly frass (BSFF) from household waste	Cameroon	BSFF improved growth parameters and yield of lettuce compared to urea and control. Application rates of 15 t·ha-1 and 30 t·ha-1 showed no significant difference	BSFF has potential as a sustainable organic fertilizer for lettuce production, but decontamination methods may be needed	Dzepe et al. (2022)

The production of the tested biomass had relied on animal-based organics (animal manures, fecal sludge, and carcass), with few such studies utilizing plant feedstock such as palm kernel meal (Hem et al., 2008) and cassava peels (Bullock et al., 2013), and coffee bean pulp (Lardé, 1990). The paradox, however is slower development of BSFL, which was evident on animal feedstock compared to plant materials (Tomberlin et al., 2005; St-Hilaire et al., 2007; Kalova, 2013). Tomberlin and Sheppard (2002) reported that growth of BSF larvae on lean decomposing pork at 27 ^°^C took 20–30 more days than their growth on decomposing grain under the same conditions. Substrate usage and suitability Major BSFL growth parameters such as development time, feed conversion efficiency, mortality, pupae weight and nutrient composition were strongly affected by the growth substrate (Zheng et al., 2013).

Optimization of production black soldier fly larvae production on organic waste for BSFL to serve effectively in the dual roles of biomass production and attendant waste reduction, establishment of a production system with a feeding strategy that specifies important substrate factors that affect BSFL feeding behavior, growth, and development is necessary (Devic and Maquart, 2015). These factors included aspects such as larvae feeding amounts (feed rate), feeding frequency (regime), substrate types and substrate combination ratios, substrate depth, larvae stocking densities, substrate moisture content, environmental rearing conditions such as temperature and relative humidity, and size of substrate particles, among others (Holmes et al., 2012). According to Diener et al. (2009), the feed consumption rate of BSF larvae depends on the size of larvae and the type of feed. The study subsequently predicted that a feeding rate of 100 mg of chicken feed at 60% moisture as the optimal tradeoff feed rate between nutrient-rich prepupa and high waste consumption in the shortest time.

### 8.5 Odor mitigation and nuisance reduction in waste handling

The sheer large numbers of BSF larvae, movement and voracious appetite enabled them to process organic waste extremely fast, aerate and dry it (Lalander et al., 2013). This prevented production of foul gases which normally occurred under anaerobic conditions and was an advantage over other methods of management such as composting (Diener et al., 2011b; Van Huis, 2013). The major benefits of BSFL are that it is useful in rapid reduction of organic waste and reduction of odor producing bacteria. Also, it competes with the house fly for growth, which helps in the prevention of several diseases spread by housefly (Kim et al., 2021). Research indicates that BSFL do not serve as vectors for human diseases, and therefore, do not pose the same nuisance as house flies (Siddiqui et al., 2022; 2024). The study by Beskin et al., 2018, resulted that BSFL reduces the level of odorous elements emitted from the animal waste. Also, it makes an alternative source for the management of animal waste which is environmentally and economically friendly (Beskin et al., 2018).

## 9 Emerging nanobiotechnological applications of BSFL systems

**FIGURE 2:**
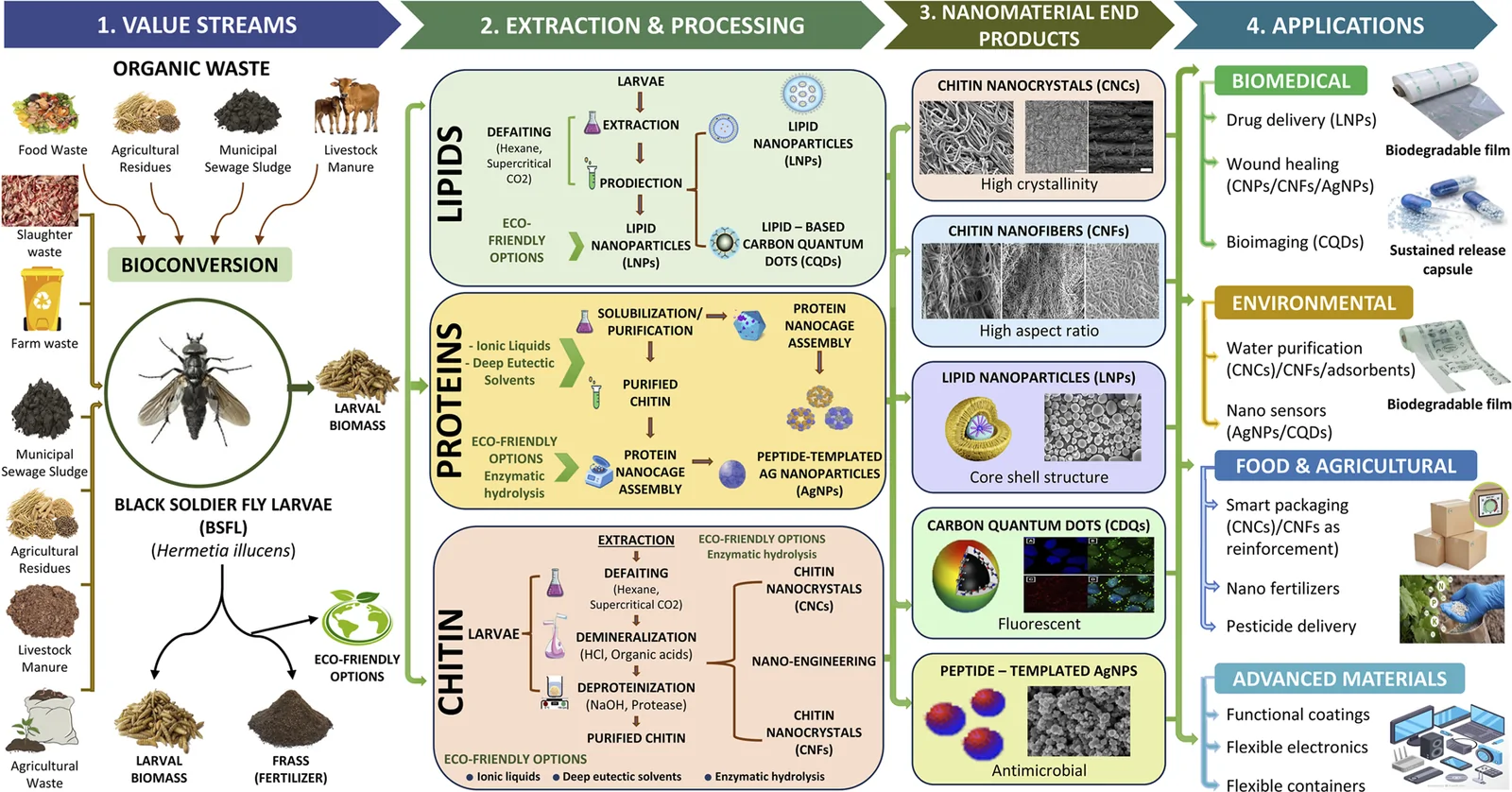
Integrated BSFL Valorization Continuum: From Waste to High-Value Nanomaterials and Applications.

The integration of nanobiotechnology into BSFL systems represents a paradigm shift from conventional waste management to development of high-performance functional materials (Figure 2). This ‘nanorefinery’ approach facilitates the recovery of high-value components from poultry waste streams that were previously unrecoverable. Other than the poultry waste, studies confirm that black soldier fly larvae (BSFL) are highly efficient bio-factories for generating diverse nanomaterials from different waste substrate. In the study by Falgayrac et al., 2024, they found that BSFL technology can be a sustainable method for bioconversion of municipal solid wastes into the chitin and chitin nanocrystal formation (Falgayrac et al., 2024). In one more study by Di Pasquale et al., 2025, they developed the protein blend nano film from the BSFL (Di Pasquale et al., 2025). Another researcher has developed the biodegradable micro/nanoplastics (BMPs) from the BSFL which can be a safer organic fertilizer production (Grossule et al., 2024).

### 9.1 Nanofabrication of chitinous scaffolds and antimicrobial agents

Dead adults and pupal exuviae BSFL serve as sustainable, non-crustacean sources of chitin (8%–24% dry weight), predominantly in the alpha-polymorphic form. A critical analysis reveals an important shift in extraction techniques from traditional using harsh chemical treatments to green nanoprocessing methods (Tan et al., 2021). For instance, Steam flash explosion (SFE) at 0.45–1.60 MPa and Natural Deep Eutectic Solvents (NADES), such as choline chloride/lactic acid, have achieved chitin purity values of 97.44%–98.58% (Zhou et al., 2019).

The use of these derived biopolymers as ‘nanowhisker’ glues and nanofibers is growing. Due to their high aspect ratio rigidity and network-forming qualities, chitosan nanowhiskers (ChNW) faclitate fatigue-resistant bioadhesion (interfacial fatigue threshold of 382 J m^-2^), thereby promoting tissue regeneration and wound healing (Jin et al., 2021). Moreover, the conjugation of chitosan derived from BSFL with selenium nanoparticles (SeNPs) and eugenol results in strong antibacterial nanocomposites that physically alter and break bacterial cell walls, including those of *S. aureus*. . In food packaging, chitosan films enriched with esterified chitin nanofibers (CN) significantly improve tensile strength and oxygen barrier properties by over five times, while also providing natural UV-blocking features (Liu et al., 2023).

### 9.2 Nano-mineral transformation and precision nano-fertilizers

Microbial-mediated nano-mineral transformation during BSFL digestion of poultry manure represents a pivotal research area. While the exact mechanisms are still being explored, the variable pH conditions present in the BSFL gut (spanning from very acidic to alkaline) and certain microbial groups (such as *Pseudomonas* species that can solubilize phosphate) are believed to aid in the precipitation of minerals and their conversion into nano-sized particles (Salahuddin et al., 2024). These procedures significantly increase the levels of calcium and phosphorus in both larvae and the resulting frass, transforming large minerals into bioavailable, frequently nanoscale types (Gil et al., 2025).

Transforming frass into nano-fertilizers tackles the shortcomings of traditional fertilizers, which often suffer from considerable drift and leaching losses. Nano-encapsulated slow-release formulations utilizing nanoporous zeolites guarantee accurate nutrient delivery and enhanced stability (Sharma et al., 2022). Recent studies reveal that bio-based precursor-derived zinc-carbon dot nano-fertilizers (Nano-ZCDs) significantly increase crop fresh weight (by about 75%) and improve soil health metrics without harming the environment (Sidhu et al., 2025). Additionally, frass supplements enriched with chitin have demonstrated the capability to boost potato production by as much as 362% while reducing soil-related diseases, including potato cyst nematodes (Anedo et al., 2024).

### 9.3 Conductive bionanocomposites in biosensing and flexible electronics

The valorization of proteins BSFL-sourced proteins, especially myofibrillar ones, presents an eco-friendly approach for creating conductive bionanocomposites in flexible electronics and biosensing uses. These proteins, plentiful in larvae raised on organic waste, create amyloid-like supramolecular aggregates through intermolecular β-sheet interactions, providing structural strength to the resultant materials (Testa et al., 2025). In addition, chitosan derived from BSFL acts as a biocompatible framework in electrochemical biosensors. Its chelating abilities allow for efficient identification of heavy metal ions like Pb^2+^ and Cd^2+^ in wastewater, aiding sustainable environmental monitoring systems. Moreover, chitosan matrices support enzyme immobilization, but observed sensitivities (such as for glutamate oxidase-based systems) correspond with typical chitosan uses rather than solely with BSFL-derived applications. The incorporation of BSFL biopolymers into cutting-edge nanocomposites illustrates a circular economy model, converting poultry waste into innovative functional materials for electronics and sensing applications (Testa et al., 2025).

Even though the BSFL have the ability to excrete specific mycotoxins and break down different pharmaceuticals, the “manure-to-nanomaterial” approach is still limited by the possible bioaccumulation of heavy metals (Cd, Pb) and enduring organic pollutants. The presence of inorganic contaminants in BSF-derived nanomaterials requires thorough characterization for their intended medical or agricultural applications. From a techno-economic viewpoint, moving to industrial-scale production necessitates the expansion of green extraction techniques like natural deep eutectic solvents (NADES) and supercritical fluid extraction (SFE), which presently involve significant capital expenses compared to conventional waste treatment methods.

## 10 Challenges and future opportunities

Leveraging Black Soldier Fly Larvae (BSFL) for sustainable poultry waste management offers a transformative approach to addressing the environmental and economic burdens of organic waste in agriculture. Poultry production generates vast amounts of manure, which, if mismanaged, contributes to nutrient runoff, greenhouse gas emissions, and pathogen proliferation (Alegbeleye and Sant’Ana, 2020). BSFL bioconversion reduces waste volume while producing protein-rich biomass for animal feed, organic fertilizer, or biodiesel, aligning with circular economy principles (Kim et al., 2021; Niranjan et al., 2024). However, scaling this technology faces technical, logistical, economic, regulatory, and social challenges that must be addressed through innovation and collaboration to unlock its full potential.

Scaling BSFL production for large-scale poultry operations is technically demanding, requiring precise environmental conditions, temperatures of 27 °C–30 °C and humidity of 60%–70%, which are challenging to maintain, especially in arid regions where water scarcity complicates humidity control (Tomberlin and Sheppard, 2002; Holmes et al., 2012). Achieving these conditions without excessive water use increases operational costs and strains scalability (Broeckx et al., 2021). Logistical hurdles include maintaining an optimal 2:1 larvae-to-substrate ratio to avoid competition and ensure efficient waste conversion (Kim et al., 2021). Excessive larval density reduces survival rates, while high moisture content in poultry manure can create anaerobic conditions, hindering degradation and frass separation (Kim et al., 2021). The variability of poultry waste, influenced by poultry diet, age, and health, further complicates industrial-scale efficiency, necessitating standardized pretreatment protocols (Sheppard et al., 2002; Kullan et al., 2024). Heavy metal accumulation (e.g., cadmium, lead) in BSFL also poses safety risks for feed applications, requiring rigorous monitoring and advanced infrastructure (Kim et al., 2021).

Economically, BSFL-derived products are cheaper than conventional feeds such as fishmeal or soybean meal due to high labor, infrastructure, and processing expenses. Despite the insect protein market’s projected growth to over $8 billion by 2030, it remains niche, and scaling production without assured demand risks financial losses (Hancz et al., 2024; Gebele et al., 2024). Regulatory variability across regions adds further complexity. Countries like China have fewer restrictions, while evolving regulations in the UK post-Brexit create investor uncertainty (Ffoulkes et al., 2021). Lengthy and costly approval processes for BSFL as animal feed, coupled with restrictions on manure-fed insects in the EU, US, and Australia, limit adoption despite BSFL’s pathogen-reducing capabilities (Ffoulkes et al., 2021; Susana, 2021; Kim et al., 2021). Public perception also poses a barrier, with concerns about microorganisms in BSFL products—though mitigated by heat treatments—slowing acceptance (Dontsop Nguezet et al., 2024).

Despite these challenges, BSFL offer significant opportunities. They use up to 95% less water than traditional livestock farming, making them ideal for water-scarce regions (Broeckx et al., 2021). BSFL reduce nitrogen by 6.1%–14.4% and phosphorus by 61%–70% in poultry waste, while producing high-protein biomass (up to 60% after defatting) for animal feed (Kim et al., 2021). This dual functionality enhances resource recovery and supports circular economy goals. BSFL treatment also lowers greenhouse gas emissions, with methane (CH_4_: 2.4 ± 0.4 mg/kg) and nitrous oxide (N_2_O: 1.0 ± 0.4 mg/kg) emissions significantly below those of composting (CH_4_: 1,500 mg/kg; N_2_O: 1,200 mg/kg) (Kim et al., 2021). These environmental benefits align with sustainability goals and strengthen the case for broader adoption.

The future of BSFL hinges on overcoming these barriers through innovation and collaboration. Technological advancements, such as automated systems for environmental control and standardized pretreatment protocols, can enhance scalability and reduce costs (Kullan et al., 2024). Crucially, the next-generation of BSFL systems must integrate nanobiotechnological innovations, such as the development of nano-biosensors for real-time monitoring of heavy metal bioaccumulation and the use of nano-encapsulated enzymes to enhance the degradation of recalcitrant manure components. Future research should prioritize the fabrication of BSFL-derived chitin nanowhiskers and carbon dots from larval frass, transitioning the system from a waste-disposal tool to a high-precision nanomanufacturing platform. Growing market demand for sustainable protein sources offers economic opportunities for early adopters (Hancz et al., 2024). Policy collaboration between governments, research institutions, and industry, supported by organizations like the FAO, can streamline regulations and build consumer confidence through harmonized standards (FAO, 2023). Public awareness campaigns highlighting BSFL’s safety and benefits can address perception barriers, drawing on the growing acceptance of insect-based feeds in Asia and Europe (Kim et al., 2021; Dontsop Nguezet et al., 2024). By addressing technical, economic, regulatory, and social challenges, BSFL can become a cornerstone of sustainable poultry waste management, driving environmental and economic benefits in a circular economy.

## 11 Conclusion

The global poultry industry, a vital pillar of food security, faces an escalating waste management crisis that conventional strategies fail to adequately address. These approaches are often energy-intensive, emit substantial greenhouse gases, and contribute to environmental pollution while offering limited resource recovery. In contrast, Black Soldier Fly Larvae (BSFL) present a scientifically validated and sustainable alternative, achieving reduction in waste dry matter, nitrogen recovery, and phosphorus sequestration, thereby significantly reducing nutrient losses and eutrophication risks. With markedly lower greenhouse gas, BSFL clearly outperform conventional waste management systems while converting poultry waste into high-value protein-rich biomass (up to 60% crude protein) and nutrient-stabilized organic fertilizer, reinforcing circular economy principles. The use of BSFL model for waste valorization proves a circular economy concept, as the process utilizes the poultry waste which can create environmental hazard and produce high protein source for the poultry itself. The waste generated at the poultry farms can be treated with the BSF and the byproduct of the techniques like insect larvae can be blend with the other poultry feed as a good protein source.

At a mechanistic level, bioconversion driven by BSFL is regulated by closely interconnected genomic, enzymatic, and microbial mechanisms, comprising of enhanced detoxification and immune gene families, specialized midgut pH variations, and cooperative interactions with a functional gut microbiome. These characteristics facilitate the effective breakdown of tough substrates like keratin, as well as successful nitrogen and phosphorus recycling. The induction of antimicrobial peptides and the concurrent reduction of antibiotic resistance genes further enhance biosecurity, contributing to safer agricultural recycling pathways. Additionally, BSFL systems demonstrate robust pathogen and pest suppression and exceptional water-use efficiency, exceeding traditional livestock production by ∼95%, strengthening their environmental and public health credentials.

Beyond waste valorization, the emerging potential to recover high-value biomolecules and nanomaterials from BSFL biomass positions this system as an advanced biorefinery platform. Synthesis of this nanobiotechnological potential reveals a transition from current achievements—focused on crude protein and lipid extraction—toward sophisticated biorefineries capable of yielding chitosan-based nanomaterials and specialized bioactive peptides. In the short-term, optimization of molecular-scale pretreatments will stabilize industrial outputs; the medium-term will likely see the integration of BSFL-derived nanoparticles in targeted agricultural and biomedical applications; and in the long-term, these systems are projected to serve as fully autonomous, high-precision biomanufacturing hubs. However, challenges related to heavy metal bioaccumulation and the absence of standardized molecular-scale pretreatment and process optimization protocols must be addressed to enable safe and large-scale industrial adoption. Future research integrating multi-omics approaches—linking transcriptomics, metabolomics, and metagenomics—will be critical for refining BSFL performance and ensuring system resilience. As global poultry production continues to intensify, BSFL-based bioconversion offers a scalable, eco-efficient, and biologically resilient pathway to bio-resource system. In future, we have to focus on the transitioning of localized waste management BSFL technique to highly automated, genetically optimized model for large scale production. Multidisciplinary research to bridge genomics, system engineering and policy making is needed to translate the BSFL technique from lab to field. Integrated approach in BSF-based technique could contribute in global circular bioeconomy.
